# Molecular Epidemiology Clinical Manifestations, Decolonization Strategies, and Treatment Options of Methicillin-Resistant *Staphylococcus aureus* Infection in Neonates

**DOI:** 10.3390/pathogens14020155

**Published:** 2025-02-05

**Authors:** Dimitrios Rallis, Natalia Atzemoglou, Konstantina Kapetaniou, Lida-Eleni Giaprou, Maria Baltogianni, Vasileios Giapros

**Affiliations:** 1 Neonatal Intensive Care Unit, School of Medicine, University of Ioannina, 45110 Ioannina, Greece; drallis@uoi.gr (D.R.); md06684@uoi.gr (N.A.); md07243@uoi.gr (L.-E.G.); mbalt@doctors.org.uk (M.B.); 2 Department of Pediatrics, School of Medicine, University of Ioannina, 45110 Ioannina, Greece; k.kapetaniou@uoi.gr

**Keywords:** multi-drug resistance, neonatal unit, *Staphylococcus*, vancomycin

## Abstract

Preterm and low-birth-weight neonates are particularly susceptible to methicillin-resistant *Staphylococcus aureus* (MRSA) colonization, whereas MRSA infection is associated with significant neonatal morbidity and mortality globally. The objective of our study was to examine the current body of knowledge about molecular traits, epidemiology, risk factors, clinical presentation, decolonization techniques, and available treatments for MRSA infection in neonates. MRSA strains that predominate in neonatal units, namely healthcare-associated (HA)-MRSA, differ from community-acquired (CA)-MRSA strains in molecular characteristics, toxin synthesis, including Panton-Valentine leukocidin, and resistance to antibiotics. Colonization with MRSA predisposes neonates to infection. The clinical impact of MRSA infection includes bacteremia, sepsis, pneumonia, endocarditis, osteomyelitis, septic arthritis, skin and soft tissue infections, and toxic shock syndrome. To reduce MRSA transmission, colonization, and infection, customized approaches are required, including continuous surveillance of MRSA epidemiology, new techniques for detecting MRSA resistance, and the application of basic preventive measures. Antimicrobial susceptibility monitoring is essential to identify the best empirical antimicrobial treatments. The growing antibiotic resistance of MRSA remains challenging, and vancomycin is still the best option. Further extensive research and surveillance are warranted to explore the genetic diversity and prevalence of MRSA.

## 1. Introduction

*Staphylococcus aureus* (*S. aureus*) is a common pathogen in humans, initially identified by Ogston [1]. *S. aureus* resistance to penicillin was first reported in the 1940s [2,3], whereas methicillin-resistant *S. aureus* (MRSA) was discovered in U.K. hospitals in 1961 [4]. A case of neonatal MRSA infection was described for the first time in the U.S. in 1981 in a neonate with osteomyelitis [5]. MRSA is a significant antibiotic-resistant strain of *S. aureus* that, currently, predominates in neonatal intensive care units (NICUs) globally and is associated with significant neonatal morbidity and mortality [6,7,8,9].

Several NICUs have reported epidemiological data on neonatal MRSA after surveillance measures, transmission control, and decolonization policies [10,11,12,13,14]. While the rate of methicillin-susceptible *S. aureus* infections was significantly reduced over the past 20 years, the rate of MRSA infections has remained consistent at about 10 per 10,000 hospitalized neonates [6]. Moreover, late-onset MRSA infections in NICUs have significantly increased by 300%, from 0.7 to 3.1 cases/10,000 days, between 1995 and 2004 [15,16]. In addition, the percentage of hospital-acquired (HA)-MRSA infections in intensive care units in the U.S. increased from 35.9% to 64.4% between 1992 and 2003 [9,17]. Nearly 1.7 million HA-MRSA infections occur each year in U.S. hospitals, including more than 33,000 cases in NICUs, according to the Center for Disease Control and Prevention (CDC) [18]. Until 2016, there had been reported more than 20 MRSA outbreaks in NICUs worldwide, most of which from the U.S. and Europe [19,20,21,22,23,24]. Recent epidemiologic studies have also reported the evolution of MRSA clones, underscoring the growing resistance of MRSA to antimicrobial agents [25]. Given these challenges, a comprehensive analysis of MRSA’s epidemiology, molecular characteristics, and toxicity is required to reduce the incidence of MRSA infections in NICUs.

**Figure 1:**
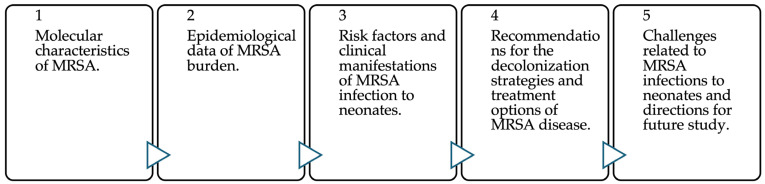
Overview of the study organization. MRSA, methicillin-resistant *S. aureus*.

Our aim was to review the existing evidence and provide novel insights into the molecular characteristics, epidemiology, risk factors, clinical manifestations, decolonization strategies, and treatment options of MRSA infection in neonates. Our study is organized into (1) exploring the molecular characteristics of MRSA, (2) providing the epidemiological data of MRSA burden, (3) reviewing the risk factors and clinical manifestations of MRSA infection in neonates, (4) providing a summary of existing recommendations for the decolonization strategies and treatment options of MRSA disease, and (5) discussing challenges related to MRSA infections in neonates and directions for future study (Figure 1).

## 2. Materials and Methods

### The Literature Search Strategy

A literature search of Pubmed was conducted by two researchers in November 2024. Only human studies and English-language articles were considered. The terms ‘Methicillin-resistant *Staphylococcus aureus*’ OR ‘MRSA’ AND ‘neonate’ OR ‘newborn’ OR ‘infant’ OR ‘Neonatal Intensive Care Unit’ OR ‘Neonatology’ were used. The retrieved studies were assessed according to their titles, abstracts, and suitability for this narrative review.

## 3. Molecular Characteristics of MRSA

HA-MRSA is transmitted within hospital settings and was formerly responsible for the majority of MRSA infections. Until the 1990s, MRSA was considered common exclusively in healthcare environments [25], but for the first time in the 1990s, MRSA was discovered in patients who had not been previously hospitalized or had any history of contact with HA-MRSA carriers [26,27,28]. Since then, epidemic outbreaks of community-acquired (CA)-MRSA infections have been reported worldwide [25,29]. The genetic investigations of these strains revealed that different strains of MRSA were present in the community compared with healthcare settings [9]. However, recent reports of neonatal MRSA infections revealed that 15–21 detected MRSA strains shared common microbiological traits with strains that have surfaced in the community, indicating that CA-MRSA has emerged in healthcare settings, including NICUs [25,29,30,31,32,33,34,35].

HA-MRSA and CA-MRSA differ from one another in terms of genotype and phenotype (Table 1) [36]. *S. aureus* genome consists of core and accessory components. The core genome includes genes that are found in every isolate and comprise nearly 75% of the *S. aureus* genome, whereas the accessory genome, which comprises nearly 25% of the *S. aureus* genome, is responsible for the large proportion of MRSA’s genetic variability. The accessory genome consists of mobile genetic elements (MGEs) that are transferred between strains, including plasmids, chromosomal cassettes, transposons, bacteriophages, and pathogenicity islands, and contains virulence, immune-evasion, and drug resistance mediators. Therefore, the accessory genome is frequently more strain-specific and varied compared to the core genome [37].

As MRSA is both a commensal and a pathogen, there is great interest in determining whether identifying MRSA colonization and attempting to eradicate carriage will lower the risk of recurrent infection. The pulsed-field gel electrophoresis (PFGE) typing system can be used for the molecular classification of MRSA strains, especially during outbreaks [32]. PFGE uses a restriction enzyme to break the bacterial DNA, which is then subjected to an electrical gradient and produces a distinctive banding pattern [38,39]. PFGE is extremely discriminatory and widely accessible; however, interlaboratory variability, technical demand, and difficulties in long-term epidemiology limit its use [40]. According to the Atlanta CDC typing scheme, most CA-MRSA infections in the U.S. have been associated with two types of PFGE, USA300 and USA400, which differ from the commonly found HA-MRSA genotypes [41]. USA300 is the most common strain related to MRSA infections in previously healthy neonates in the U.S. and Europe, and it is produced when an ancestral sequence type (ST) 8 strain absorbs an element that catabolizes arginine, the staphylococcal cassette chromosome (SCCmec) type IV, and a Panton-Valentine leukocidin (PVL)-encoding locus [21,42,43]. The arginine-catabolic element increases the ability of the USA300 MRSA strain to elude the immune system and survive inside the host, while SCCmec confers antibiotic resistance and PVL increases invasiveness [32,44]. There have been prior reports of USA400-caused outbreaks of skin and soft tissue infections (SSTIs) in term neonates associated with transmission in neonatal nurseries and postnatal wards [45,46]. Finally, the gene locus *sasX* has been recently detected in MRSA clones such as ST5 and may be involved in nasal colonization, lung infections, and the development of abscesses [7,47].

The supplementation of PFGE with multilocus sequence typing (MLST) provides additional comparisons with sequences described in available databases. MLST is highly repeatable, discriminate, and appropriate for long-term worldwide epidemiology. A standardized MLST database defines each strain’s alleles, while allele combinations identify strains [48].

*Spa* typing is based on sequence-based analysis of the sequence region of the *spa* gene of polymorphism X. It is rapid and ideal for outbreak investigations. References to extensive foreign databases are made to the sequences [49]. The multilocus variable number of tandem repeat analysis evaluates the variation in the number of repeat DNA sequences, and it is high-throughput and low-cost [50].

SCCmec typing is based on PCR and assigns distinct SCCmec types to specific allotypes of the *mecA* and *ccr* genes. Compared to whole-genome sequencing or MLST, it is less expensive. Divergent and developing SCCmec types that are not detectable by present approaches have been found using SCCmec typing to MRSA [51]. Types that are more prevalent in hospital or community settings can be identified by using the SCCmec typing [32]. HA-MRSA strains have been associated with SCCmecA types I–III, while CA-MRSA strains have been associated with SCCmecA types IV, V, and VII [8,36]. These different SCCmecA types carried by HA-MRSA or CA-MRSA strains provide distinct patterns of antibiotic resistance by encoding the penicillin-binding protein 2A (PBP2A) [36]. Unlike the HA-MRSA phenotype, CA-MRSA is susceptible to various antibiotics, including clindamycin, quinolones, and trimethoprim-sulfamethoxazole [8,52,53,54].

Repetitive element palindromic PCR is a genotyping method that identifies repetitive DNA sequences dispersed across the MRSA genome. STAR gene restriction profile analysis is based on PCR amplification and restriction enzyme digestion and generates restriction profiles that differ according to the intergenic regions’ sequence within the PCR product [55].

Finally, whole-genome sequencing analyzes the entire genome sequence for single-nucleotide variants [56]. The resolution offered by whole-genome sequencing has allowed us to determine that individuals who were colonized by circulating community strains later introduced those strains into hospitals, which ultimately led to the intermixing of CA-MRSA and HA-MRSA strains [57]. MGEs and essential genome components were discovered in later efforts to define the elements that contributed to those strains’ success.

Differences in phenotypic characteristics between HA-MRSA and CA-MRSA are also underscored by the carriage of PVL and the greater expression of additional toxins in CA-MRSA compared to HA-MRSA strains [32,58]. Epidemiologic studies during the last decade revealed that specific clonal lineages, namely ST 1, 5, 8, and 22, are responsible for most neonatal MRSA infections globally [20,21,22,25,31,33,43,59,60,61,62,63,64]. Many of these clones, such as ST8 and ST22, the two most prevalent MRSA clones in the U.S. and Europe [65], are characterized by genetic plasticity and hold the ability to produce toxins and biofilms [66]. Collagen-binding proteins, fibronectin, elastin, and clumping factors all contribute to MRSA’s ability to aggregate on host tissues and indwelling devices [66]. Among several MRSA-produced toxins, PVL leukotoxins, LukED, LukAB/DH, hemolysins, exfoliative toxins, enterotoxins, phenol-soluble modulins, and toxic-shock syndrome toxin-1 are the most important [67]. While it remains unclear how the neonatal immune system reacts to MRSA infection [68], the production of toxins has been related to the generation of proinflammatory cytokines leading to hyperinflammation and tissue injury [65,69,70,71]. This unbalanced inflammation is also considered the major underlying mechanism associated with long-term neonatal morbidities, such as cerebral palsy, retinopathy of prematurity, necrotizing enterocolitis, and bronchopulmonary dysplasia [7].

### Antimicrobial Resistance

Clinical diagnosis now relies on the availability of sensitive and precise techniques for accurately identifying antibiotic resistance in MRSA. Molecular typing is warranted because phenotypic typing techniques are extremely reliant on growth conditions and are not capable of reliable discrimination [72,73]. MRSA has acquired MGEs, including insertion sequences, transposons, and, occasionally, plasmids containing genes for antibiotic resistance to penicillin (*blaZ*), erythromycin (*ermC*), clindamycin (*ermC*), trimethoprim (*dfrA* and *dfrK*), and tetracyclines (*tetK* and *tetL*) [74].

The *mecA* gene, which codes for PBP2A, may be a useful molecular marker of MRSA [75]. The resistance to methicillin is mainly attributed to the overexpression of PBP2A, which has a low affinity for β-lactam antibiotics; nevertheless, other mechanisms, such as efflux pumps, are also associated with methicillin resistance [76]. Antibiotic resistance in HA-MRSA strains is also genetically associated with resistance in disinfectants or heavy metals such as quaternary ammonium, mercury, or cadmium, probably reflecting the high selection pressures present in the hospital setting [77].

Vancomycin-resistant *S. aureus* (VRSA) was initially detected in Japan in 1996, but numerous reports thereafter indicate that it has since spread. Given the extensive use of vancomycin to treat MRSA infections, the most worrisome genetic adaptation in *S. aureus* to date is the development of resistance to this antibiotic. There are two types of vancomycin resistance in *S. aureus*. Long or multiple courses of vancomycin often result in the emergence of vancomycin-intermediate *S. aureus* (VISA) strains. Several distinct mutations within a population result in varying levels of vancomycin resistance. The majority of mutations found in VISA isolates change essential genomic elements involved in cell wall production and autolysis. Cross-resistance to daptomycin is also conferred by a number of these mutations, such as those in *yycH*, *mprF*, and *dltA*. Unlike VISA, it has been demonstrated that plasmid transfer of the vanA operon from vancomycin-resistant *Enterococcus faecalis* results in VRSA [78]. Complete resistance to vancomycin is achieved when vancomycin molecules are trapped due to the thicker cell wall and obstructing the peptidoglycan plexus, which serves as a physical barrier against vancomycin molecules.

## 4. Epidemiology of MRSA Burden

Over the past few decades, there has been a conceptual evolution in the epidemiology of *S. aureus* [7]. Hospitalized neonates have a higher rate of MRSA colonization than the general neonatal population, with rates varying from 0.3% to 32% among institutions [22,23,31,33,60,79,80,81], explained by the 2–3 times higher MRSA carriage rate among healthcare workers compared with the general population [24,54,59].

In a previous systematic review based on studies from high-income countries in Europe and the Western Pacific Region, the pooled prevalence of MRSA carriage was 9.5% among healthcare providers [82]. Similarly, another systematic review in South Asia reported an MRSA carriage rate in healthcare providers at 9.23% [83]. Of note, a systematic review of 22 studies in Iran reported a much higher rate of 32.8% of nasal MRSA carriage among healthcare providers [84]. Individual studies reported a prevalence of MRSA colonization in healthcare providers of 27.91% in Jordan [85], 26.47% in India [86], 17.65% in Nigeria [87], and 16.22% in Greece [88].

The prevalence of nasal MRSA colonization in neonates usually ranges from 2% to 4%, but it might reach up to 8% during an MRSA epidemic investigation [8,13,14,33,89]. In addition, previous studies have found that between 0.6% and 8.4% of neonates were colonized or infected with MRSA during respective periods [11,14,43,90,91,92,93]. According to Huang et al., MRSA colonized 5.2%, methicillin-sensitive *S. aureus* colonized 12%, and *S. aureus* colonized 17% of the neonates’ nasal cavity overall [91]. In a previous study in China that evaluated the burden of *S. aureus* at the time of admission to the NICU, 17% of neonates had nasal colonization with MRSA or methicillin-sensitive *S. aureus* at the time of admission [81] compared to 13% of neonates in a NICU in Taiwan (13%) [31], 10% in Japan [94], and 3.8% in the U.S. [80]. According to a systematic review of 62 studies conducted between 2001 and 2023 in the U.S., Japan, South Korea, Brazil, Taiwan, and other countries, the Western Pacific region had the highest rate of MRSA colonization (19.8%), whereas America had the lowest (3.1%) [95]. A cumulative incidence of 1.4% to 3% was observed for CA-MRSA infections, while a cumulative incidence of 9.5% to 9.8% was observed for HA-MRSA infections. There were regional differences, with Taiwan having the highest prevalence at 23.8% and Brazil having the lowest at 0.9%. Compared to the U.S., South Korea had a greater HA-MRSA (21.9% compared to 2.9%) and CA-MRSA incidence (8.5% compared to 1.5%) [95]. The yearly incidence rate of MRSA colonization in China varied between 5.66 and 7.66 cases per 1000 admissions [96]. According to data from 33 centers across 11 Latin American nations for the Tigecycline Evaluation and Surveillance Trial, the total prevalence of MRSA among *S. aureus* isolates was 48.3% between 2004 and 2007 [97]. The SENTRY Antimicrobial Surveillance Program in Latin America also revealed that the frequency of MRSA among staphylococcal infections in medical centers increased from 33.8% in 1997 to 40.2% in 2006 [98]. Finally, a recent meta-analysis by Zervou et al. reported that the pooled prevalence of MRSA colonization was 2.3%, based on 11 studies that were conducted in the U.S., compared to 1.3% based on studies conducted in Asia [89]. The prevalence of MRSA colonization on admission was 1.5%; interestingly, the prevalence of MRSA colonization in outborn neonates was 5.8% compared with 0.2% in inborn neonates.

The incidence of invasive *S. aureus* infections was more than 25% in 8 out of 30 European countries [99]. Romania, Malta, Portugal, Cyprus, Greece, Italy, Slovakia, and Spain reported an incidence of >25%, while Hungary, Croatia, and Ireland had an incidence higher than the European Union population-weighted mean of 16.8% [99]. According to previous reports, MRSA was responsible for 33–67% of *S. aureus* infections in neonates, and among neonates with MRSA colonization, one-fourth developed MRSA infections [33,43]. During a 20-year study period in Western Australia, *S. aureus* sepsis was responsible for about 4% of blood culture-positive infections in neonates, with an overall incidence of 0.10/1000 live births. Infants born before 32 weeks of gestational age had a much greater incidence of *S. aureus* sepsis (6.87/1000 live births) than infants born after 32 weeks of gestational age (0.03/1000). Between 2001 and 2010, the frequency of *S. aureus* sepsis was 0.13 per 1000 live newborns, and between 2011 and 2020, it was 0.07 per 1000 live births. MRSA was responsible for 26% of cases, whereas methicillin-sensitive *S. aureus* was responsible for 74% [100].

## 5. Risk Factors and Clinical Manifestations of MRSA Infection in Neonates

### 5.1. Colonization

Neonates in the NICU are a particularly vulnerable population. Specific innative and environmental factors, such as the immature neonatal immune system, exposure to multiple invasive procedures, prolonged hospitalization, and close contact with healthcare providers, predispose neonates to MRSA colonization and infection [12,101,102,103].

Neonates may acquire *S. aureus* through the birth canal [104,105], while a concurrent maternal infection may be present in up to 20% of newborns with MRSA infection [43]. The rate of vaginal MRSA colonization among pregnant women has been estimated to be 2.8% in previous reports [106], and vaginal delivery has been associated with increased risk of *S. aureus* neonatal transmission [81]. In a previous systematic review, the pooled proportion of MRSA carriage among neonate mothers was 2.1% [82], with studies from Jordan reporting a prevalence of 9.72% MRSA colonization [85], Egypt of 1.69% [107], Brazil of 1.39% [108], and Germany of 0.51% [109]. Vertical transmission of MRSA has also been indicated by the association of maternal MRSA chorioamnionitis with neonatal MRSA sepsis [110]. Female sex and multiple gestation are additional risk factors for MRSA colonization and infection [81,91,111], whereas antibiotic administration in the week before delivery has been associated with a lower risk of MRSA transmission [81].

After birth, neonates are exposed to *S. aureus* following contact with adult skin [112,113]. The carriage of *S. aureus* among adults ranges from 30% to 70%. MRSA has been shown to spread horizontally through contact with healthcare providers or the hospital setting [46,114,115]. Other factors, including NICU overcrowding and understaffing, have been associated with a higher risk of colonization and transmission and could result in MRSA outbreaks [102,103]. It has also been demonstrated that mothers can vertically transmit MRSA to their infants through breast milk [116,117], while fathers can through direct contact with their infants [118]. Controlling transmission is more challenging in intensive care units because *S. aureus* can persist on ambient surfaces for extended periods [119].

Among neonatal factors, prematurity and low birth weight are the main risk factors for MRSA colonization and infection [15,31,60,120]. Numerous studies have shown that low birth weight was associated with an increased risk of MRSA colonization and/or infection [90,91,93,111,121]. The prevalence of MRSA infection in extremely low-birth-weight neonates was estimated at 53.4 per 10,000 infants, which was much higher compared to 23.2, 7.9, and 5.0 per 10,000 infants in very-low-birth-weight, low-birth-weight, and appropriate-birth-weight neonates, respectively [15]. Neonates of lower gestational age were also more susceptible to being positive in nasal or both nasal and groin MRSA colonization, compared to neonates of higher gestational age who were positive on groin swabs only [122].

Long-term ventilation support, intravascular catheters, antibiotic administration, total parenteral nutrition, and surgical interventions are additional risk factors for MRSA infections [7,22,33,60]. In the NICU, neonates frequently need several procedures during their hospital stay, including endotracheal tube insertion, mechanical ventilation, central vascular catheterization, and surgery [90]. A previous study demonstrated that compared with non-MRSA-colonized infants, MRSA-colonized infants who experienced a greater incidence of late-onset sepsis were more likely to be intubated or mechanically ventilated [16]. A recent review of *S. aureus* outbreaks in neonatal intensive care units conducted in Leeds, U.K., identified that MRSA bacteremia was more likely to occur in infants with discharge skin lesions, prior abdominal surgery, current MRSA colonization, and Broviac or peripherally implanted central catheter lines [119]. An increased risk of MRSA infection has also been associated with feeding practices such as parenteral nutrition [121] and gavage feeding [111]. Moreover, longer hospital stays [111] and kangaroo care [93] were other independent risk factors for MRSA infection.

Among all risk factors, MRSA colonization is the most significant risk factor for developing MRSA infection in neonates. According to Huang et al., MRSA-colonized neonates had a considerably greater rate of MRSA infection (26%) than non-colonized neonates (2%) [91], whereas, as suggested by the metanalysis by Zervou et al., colonized neonates have a 24.2-fold higher chance of contracting an MRSA infection while in the NICU compared with non-colonized newborns [89].

### 5.2. Clinical Manifestations

Neonates may be colonized with MRSA within a median of 9 days from admission, with a range of 1–91 days [22]. In addition, the median interval between MRSA colonization and infection is 4 to 9 days [33,79]. In a previous report from a New York NICU, MRSA colonization was detected at a median of 17 days, with a range of 4 to 159, whereas nearly two-thirds of the neonates developed colonization during the first 3 weeks of life [16].

MRSA infection has a significant clinical impact. The most common manifestations of MRSA infections in neonates are SSTIs; however, invasive diseases have also been reported [9,42,123,124,125,126]. Most cases of invasive MRSA disease (75%) are associated with bacteremia [18]. Late-onset sepsis is a common clinical manifestation of MRSA infection and can range from a moderate focal infection to severe invasive disease [119,127,128]. According to reports, late-onset newborn sepsis can raise mortality from 7% to 18% and lengthen the inpatient stay by three weeks [119]. Among neonates with *S. aureus* bacteremia in a ten-year retrospective research in the U.K. between 1993–2003, MRSA was detected in nearly one-third of neonates [129]. Similarly, among neonates with *S. aureus* bacteremia in the U.S., MRSA was found in 47% of the cases, indicating that MRSA has emerged as a major cause of neonatal sepsis [35]. Finally, Dolapo et al. reported that the prevalence of MRSA bloodstream infections in neonates increased from 24% to 55% between 2000 and 2009 [130].

Infectious endocarditis, abscesses in the myocardium, liver, spleen, or kidneys, necrotizing pneumonia, osteomyelitis, myositis, meningitis, toxic shock syndrome, septic thrombophlebitis, venous thrombosis, sustained bacteremia, ocular infections, and Waterhouse-Friderichsen syndrome are just a few of the numerous MRSA manifestations that have been reported [131,132,133,134,135].

CA-MRSA infections typically manifest as SSTIs, in contrast to HA-MRSA infections, although more severe invasive manifestations can also occur [136]. In comparison with HA-MRSA, CA-MRSA contains the virulence genes lukS-PV/lukf-PV that generate PVL, and produce a pore-forming cytotoxin that causes leukocyte death and tissue necrosis [137]. In the U.S., CA-MRSA was the most common cause of SSTIs [123,124,125]. The clinical manifestations of SSTIs can vary from cellulitis or a simple abscess to more serious soft-tissue infections such as necrotizing fasciitis, pyomyositis, and mediastinitis as a consequence of retropharyngeal abscess [138,139,140,141]. When term neonates have localized only pustulosis with no signs or symptoms of sepsis, lumbar puncture is not required [126].

Careful patient monitoring and prompt access to microbiological and laboratory tests are crucial because the clinical symptoms and indicators at the beginning of MRSA infections can be non-specific [120]. In addition, MRSA-infected newborns may have a higher readmission rate and a longer course of infection than methicillin-susceptible *S. aureus* cases [30,142]; however, there appears to be no difference between MRSA and methicillin-susceptible *S. aureus* in terms of clinical presentation and mortality [6,120,143]. In very immature preterm neonates, MRSA infections increase the risk of unfavorable short- and long-term outcomes, as well as mortality [144,145,146]. The mortality rate of MRSA infections ranges from 2.9% to 28%, with significant variation across institutions [6,20,60,120]. According to earlier research, the case fatality risk of neonatal MRSA sepsis ranged between 9.5% and 55% [147]. A previous study also reported that among MRSA infections, sepsis had a mortality rate of 16%, pneumonia of 32.1%, and necrotizing enterocolitis of 27.3% [148].

## 6. Decolonization Strategies and Treatment Options for MRSA

### 6.1. Precautions Against Colonization

Neonates are colonized when passing through the maternal birth canal. Moreover, newborns who are placed on the mother’s breast as soon as possible after delivery are colonized with the maternal skin microbiome. *Neisseria* and *Streptococcus* species are two of the many bacteria that quickly colonize a newborn’s mouth. According to Fukuda et al., newborns who were breastfed exhibited a quick rise in common α or Á-*Streptococcus* in their mouths [149]. Importantly, Uehara et al. showed that precolonization of neonatal mouth and nostrils with common α- and/or Á-*Streptococcus* prevented MRSA colonization [150]. Additionally, distributing the mother’s breast milk over and into the mouths of extremely-low-birth-weight neonates as soon as they are admitted into the NICU can greatly reduce the colonization rate of MRSA in their mouths [148].

The most crucial infection control measure is strict hand hygiene before and after handling neonates; however, this is one of the least followed. Hand hygiene using tap water alone can significantly reduce the risk of infection, even in the absence of a disinfectant. Nonetheless, the use of chlorhexidine gluconate and other similar disinfectants in soap is not an efficient preventive measure and is only as effective as using tap water because many strains of MRSA are resistant to these disinfectants. Research has demonstrated that the MRSA isolation rate decreases when gloves are used as an infection control method when handling neonates [148,151]. An overall guidance for precautions against MRSA colonization is depicted in Table 2.

### 6.2. Decolonization

Currently, prevention rather than treatment is the best approach to managing neonatal MRSA infections. Preventing MRSA transmission in the NICU is essential because MRSA colonization is the major risk factor for developing MRSA infection [12,91]. Strict hand hygiene is crucial in preventing MRSA spread, in addition to surveillance and decolonization [152]. Cohorting and isolating MRSA-positive patients, taking barrier precautions, educating healthcare professionals, avoiding crowded wards, and monitoring and decolonizing parents and healthcare providers are additional strategies that may prevent MRSA infections [22,24,59,121,152,153].

Several NICUs have implemented detection and isolation programs to prevent the spread of MRSA and decrease infection rates. These programs employ surveillance to promptly identify affected patients, followed by cohorting and isolation using standard contact precautions. Decolonization is the key to preventing infection. NICUs have reported varying degrees of success following policies of active MRSA surveillance swabs and decolonization using nasal mupirocin with or without an antiseptic [7]. Controlling MRSA transmission in NICUs is challenging because healthcare providers, parents, family members, and visitors are asymptomatically colonized and unintentionally act as reservoirs for transmission [154]. Furthermore, *S. aureus* lives on environmental surfaces for extended periods [155]. According to the CDC 2021 *S. aureus* NICU recommendations, NICU patients should at minimum have their anterior nares swabbed [155]. The recommendation that the umbilicus and neck be expressly listed as preferred screening sites in neonates has been deleted from the recently updated National Institute of Clinical Excellence guidelines on the management of MRSA due to a lack of evidence [156].

However, targeted MRSA decolonization techniques may have limitations. First, up to 42% of infected neonates have no previous positive MRSA screening swab, preventing any chance of decolonization, even with weekly monitoring cultures [13]. Second, the median period between colonization and infection is only 5 days, which reduces the window of opportunity for decolonization for many neonates. Third, the effectiveness of decolonization to eliminate MRSA colonization and prevent MRSA infections may be restricted because, according to previous reports, 38% of neonates who had decolonization treatment became recolonized during their NICU stay, and 16% contracted an MRSA infection [102]. To effectively reduce MRSA infections in neonates, some authors have suggested treating all newborns with mupirocin [6]. Many NICUs have established protocols to identify and isolate colonized children since MRSA-colonized infants frequently act as a reservoir for transmission to other infants [13,157]. Unknown is how treating all newborns, including those that are not colonized, may change the neonatal microbiome over time. It is noteworthy that, in some situations, a universal approach has led to the development of mupirocin resistance [158]. It has been noted that controlling MRSA outbreaks in NICUs can be also challenging [91,154]. Such outbreaks have only been successfully contained by the application of strict infection control measures, sometimes in conjunction with mupirocin treatment. Decolonization methods (Table 3) in addition to continuous reinforcement of hygienic measures should include (1) mupirocin twice a day for 5 to 10 days to decolonize the nasal cavity and (2) topical body decolonization regimens using a skin antiseptic solution, such as chlorhexidine, for 5–14 days.

### 6.3. Antimicrobial Therapy

To obtain the best empirical antimicrobial treatments for neonates suspected of having an MRSA infection in NICUs, antibiotic susceptibility monitoring is essential. According to several studies, individuals with MRSA bacteremia may benefit from taking a beta-lactam in addition to vancomycin or daptomycin to reduce the duration of their illness and prevent recurrence [159]. Although the majority of MRSA isolates were susceptible to trimethoprim-sulfamethoxazole, tetracycline, rifampin, linezolid, ceftaroline, chlorhexidine, and mupirocin, surveillance studies over the past decade have revealed high resistance rates to erythromycin, clindamycin, and ciprofloxacin [16,33,160].

An overview of the treatment options for MRSA infection is depicted in Table 4. In term neonates, topical mupirocin therapy may be sufficient for minor cases of localized pustulosis [161]. Until bacteremia is ruled out, vancomycin or clindamycin should be used in cases of localized diseases in premature or very-low-birth-weight neonates or more widespread diseases affecting many sites in term infants [126].

The best treatment for severe MRSA infections in newborns is vancomycin, although numerous antibiotics have been explored with varying degrees of efficacy [7,162]. The use of combination therapy with rifampin, gentamicin, or daptomycin in neonatal sepsis should be decided on an individual basis because there is little evidence of its possible benefits [161]. There have been reports of VISA, and even VRSA, strains, that have acquired vanA resistance from strains of vancomycin-resistant *enterococci* [47,163]. Since vancomycin is the empirical antibiotic of choice for neonates with sepsis and extensive skin infections, particularly in areas with high MRSA prevalence, its decreased susceptibility to MRSA presents a significant challenge [161,164]. Strategies that target the virulent determinants of MRSA may show promise, although their effectiveness and safety in neonatal populations have not yet been confirmed [67].

There is limited experience using clindamycin and linezolid for severe MRSA infections in neonates; however, these medications may be used to treat neonates with susceptible isolates who have non-endovascular infections [161,165,166]. The U.S. Food and Drug Administration (FDA) has approved clindamycin for the treatment of severe *S. aureus* infections. It has gained widespread use for treating SSTIs and has been effectively used to treat invasive susceptible CA-MRSA infections in children, including osteomyelitis, septic arthritis, pneumonia, and lymphadenitis, despite not being specifically approved for the treatment of MRSA infections [166,167,168,169]. It is not recommended for endovascular infections such as septic thrombophlebitis or infective endocarditis because of its bacteriostatic properties. Although its entry into the cerebrospinal fluid is restricted, clindamycin has exceptional tissue penetration, especially in bone and abscesses [161].

Linezolid is a synthetic oxazolidinone that prevents the 50S ribosome from initiating protein synthesis. The FDA has approved this treatment for nosocomial MRSA pneumonia and SSTIs in adults and children. It is also in vitro active against VRSA and VISA [170,171]. Although an outbreak of MRSA infection resistant to linezolid has been reported, linezolid resistance is uncommon [172]. Long-term use usually results in resistance through a mutation in the 23S ribosomal RNA-binding site for linezolid or methylation of adenosine at position 2503 in 23S ribosomal RNA caused by the *cfr* gene [173,174].

Daptomycin is an antibiotic of the lipopeptide class that causes bactericidal action in a concentration-dependent manner by interfering with the function of cell membranes through calcium-dependent binding. Research is ongoing to establish the pharmacokinetics, safety, and efficacy of daptomycin in children [175]. Due to a lack of research on daptomycin’s effectiveness and safety, it is not frequently used in neonates, with numerous examples, despite having demonstrated the advantages and relative safety of daptomycin use in newborns [176,177]. When vancomycin fails clinically, daptomycin may be considered. Due to their synergistic impact, daptomycin and beta-lactams are more successful when used in combination to treat invasive MRSA infections, including bacteremia and endocarditis [178,179,180]. However, according to a randomized clinical trial conducted between 2015 and 2018, there was no correlation between beta-lactam use and lower treatment failure and death when used in combination with regular vancomycin or daptomycin therapy [181], while a meta-analysis suggested that combined treatment might enhance certain microbiological outcomes but not mortality [182].

Rifampicin exhibits bactericidal action against *S. aureus*, reaches high intracellular levels, and penetrates biofilms [183]. It should not be used as monotherapy due to the rapid development of resistance; however, in some situations, it may be used in combination with another active antibiotic.

Telavancin, a parenteral lipoglycopeptide, prevents cell wall formation by attaching to peptidoglycan chain precursors and depolarizing cell membranes [184]. MRSA, VISA, and VRSA are all susceptible to its bactericidal effects.

The FDA has not approved trimethoprim-sulfamethoxazole for the treatment of staphylococcal infections. However, trimethoprim-sulfamethoxazole has emerged as a significant option for the outpatient treatment of SSTIs as 95–100% of CA-MRSA strains are sensitive in vitro [136,185]. Because trimethoprim-sulfamethoxazole increases the risk of kernicterus, it is not recommended during the first few months of life.

## 7. Discussion

Over the past 40 years, MRSA has become a significant pathogen that has spread to hospitals and the community. It is the primary cause of HA infections, including bacteremia, endocarditis, SSTIs, and infections of the bones and joints [186]. Although the prevalence of MRSA has decreased, it still poses a serious clinical risk; hence, special attention is required. Routine surveillance and accurate detection of MRSA strains are crucial for providing the best antibiotic therapy, comprehending the evolution of nosocomial transmission control, and implementing preventative measures. Furthermore, public health in Europe continues to prioritize *S. aureus* or MRSA, as evidenced by the fact that 8 out of 30 countries, including Greece, report prevalence rates of MRSA > 25% [99]. The significant increase in MRSA colonization upon admission may support some centers’ practice of isolating their outborn population until their MRSA status is determined, even though the CDC does not list interhospital transfer of neonates as one of the clinical conditions for transmission-based precautions [79,187]. Significantly, compared with non-colonized neonates, those who are MRSA carriers upon admission to the NICU have a significantly higher risk of contracting an MRSA-associated infection while in the hospital.

To reduce MRSA rates and reduce disease transmission, numerous NICUs have implemented active detection and isolation programs [16]. Attempts have been made in various healthcare settings to implement universal MRSA-targeted decolonization. Because strains that colonize neonates and cause subsequent infections are strongly associated, many NICUs have attempted either targeted or universal decolonization as a method of preventing MRSA infections [91,157,188]. However, the results of these policies have been inconsistent, with the development of resistance being among the possible drawbacks. Moreover, previous studies have demonstrated that several MRSA strains can be detected in NICUs [13], with Carey et al. reporting that several strain types were detected in colonized/infected neonates over eight years, even though data from routine weekly surveillance cultures were lacking [90]. Larger studies are required to ascertain the cytotoxicity status of *S. aureus* to better understand whether these are potentially useful markers to take into consideration in future decolonization programs, given recent evidence regarding the potential role of virulence ascertained using comparable in vitro assays [81].

A shift in epidemiology, with CA SCCmec genotypes becoming more and more linked to hospital infections, was indicated by the SCCmec typing results, which showed a mix of CA-MRSA and HA-MRSA genotypes in the hospital [189]. After Healy et al. published the first report of CA-MRSA infections in NICU patients in 2004, similar changes from HA-MRSA to CA-MRSA strains were observed in additional NICUs [16,35].

Mupirocin decolonization works effectively and has little adverse effects in MRSA-colonized neonates [190]. Parental decolonization is another tactic that has recently been assessed to reduce neonatal MRSA colonization and subsequent infection. Decolonization of *S. aureus*-colonized parents reduced the incidence of infants acquiring an *S. aureus* strain concordant with a parental strain by 57%, according to a previous randomized controlled trial conducted in the U.S. [154]. Strict commitment to neonatal decolonization methods combined with parent decolonization may be required to decrease infant colonization and infection [191].

To identify the best empirical antimicrobial treatments for patients with suspected infections, antibiotic susceptibility monitoring in individual NICUs is essential. Vancomycin is still the best option for treating MRSA infections, although VISA and VRSA have emerged as examples of MRSA strains that are vancomycin-resistant. The co-occurrence of MRSA and VRSA phenotype has been reported in previous studies, in Asia, Europe, and North America, raising significant concerns. Human-origin isolates showed a susceptibility trend, indicating that linezolid should be the final medication of choice for multidrug-resistant MRSA. Additionally, human infections are increasingly related to oxacillin-susceptible MRSA [192,193,194]. Traditional susceptibility testing may mistakenly identify oxacillin-susceptible MRSA strains as methicillin-sensitive *S. aureus*, making it more difficult to diagnose and treat *S. aureus* infections, underscoring that public health should prioritize surveillance of such new pathogens.

## 8. Conclusions

The higher survival rate of very immature preterm neonates has led to an increase in the number of newborns at risk for MRSA colonization and infection. Despite the abundance of data reported from NICUs worldwide, multicenter or population-based studies to elucidate the epidemiology and clinical features of neonatal MRSA colonization and infections are lacking. Such information is essential for precisely estimating the MRSA disease burden and supporting surveillance and preventative decision-making.

MRSA transmission, colonization, and infection in the NICU are complicated issues. The significance of reducing the colonization rate in the NICU is highlighted by the 24.2 relative risk of recurrent infection among MRSA carriers compared with non-carriers. To reduce MRSA colonization, infection, and transmission in hospitalized neonates, customized approaches are required. Data from prospective randomized multicenter trials and continuous local surveillance of clinical and molecular epidemiology of MRSA must be combined to effectively control MRSA in the NICU. It is crucial to address the rapid changes in MRSA population structure and pathogenic factors; therefore, new techniques for detecting MRSA resistance are required. Because of the increasing antibiotic resistance of MRSA and the uncertainty surrounding the safety and efficacy of decolonization procedures in newborns, basic preventative measures continue to be the key to reducing neonatal MRSA infections. New strategies to limit MRSA from endangering NICU patients should be developed, including molecular analysis of the strains, shifting patterns of antibiotic susceptibility, and the existence of possible virulence factors. Further extensive research and surveillance are warranted to explore the genetic diversity and prevalence of MRSA.

## Figures and Tables

**Table 1:** Differences between HA-MRSA and CA-MRSA.

	HA-MRSA	CA-MRSA
SCCmec.	Types I, II, III.	IV, V, VII.
PFGE typing.		USA300, USA400.
Sequence types.	1, 5, 8, 15–21, 22.	5, 8, 239.
Toxin carriage.		Panton-Valentine leucocidin.
Clinical manifestations.	Bacteremia, sepsis, endocarditis, pneumonia, osteomyelitis, septic arthritis, central nervous system infections.	Skin and soft tissue infections, toxic shock syndrome.
Antibiotic susceptibility.	Vancomycin, linezolid, daptomycin, telavancin.	Clindamycin, quinolones, trimethoprim-sulfamethoxazole, vancomycin, linezolid, daptomycin, telavancin.

HA-MRSA, hospital-associated methicillin-resistant *S. aureus*; CA-MRSA, community-acquired methicillin-resistant *S. aureus*; SCCmec, staphylococcal cassette chromosome; PFGE, pulsed-field gel electrophoresis typing.

**Table 2:** Precautions against MRSA colonization.

Policy	
Nasal and throat bacterial flora.	Placement on the maternal breast as soon as possible after delivery. Precolonization of the common α- and/or Á-*Streptococcus* by distributing the mother’s breast milk over and into the mouth of extremely-low-birth-weight neonates as soon as they are admitted into the NICU.
Bacterial flora of the skin.	Skin-to-skin contact between the newborn and the mother should be established in the delivery room as soon as possible following birth, regardless of the mode of delivery. Kangaroo care.
Hand hygiene.	Strict hand hygiene before and after handling neonates.
Wearing gloves.	MRSA isolation rate decreases when gloves are used as an infection control method.
Avoid overcrowding/Cohorting	Cohorting and isolating MRSA-positive neonates, taking barrier precautions, educating healthcare professionals, and avoiding crowded wards.

MRSA, methicillin-resistant *Staphylococcus aureus*; NICU, neonatal intensive care unit.

**Table 3:** Measures for MRSA decolonization.

	Indications	Limitations
Mupirocin nasal.	Twice a day for five to ten days to decolonize the nasal cavity.	42% of infected neonates had no previous positive MRSA screening swab. Many newborns had a small window of opportunity for decolonization because the median time between colonization and infection was only 5 days. The effectiveness of decolonization to eliminate MRSA colonization and prevent MRSA infections may be restricted because several neonates who had decolonization treatment became recolonized during their NICU stay and a few contracted an MRSA infection.
Chlorexidine antiseptic solution.	Topical body decolonization regimens using a skin antiseptic solution, such as chlorhexidine, for 5–14 days.	

MRSA, methicillin-resistant *Staphylococcus aureus*; NICU, neonatal intensive care unit.

**Table 4:** Antimicrobial therapy against MRSA.

	Indications	Limitations
Mupirocin.	Topical therapy may be sufficient for minor cases of localized pustulosis. Mupirocin binds to bacterial isoleucyl-transfer-RNA synthetase, selectively and reversibly. When this enzyme is inhibited, bacterial protein and RNA synthesis are inhibited.	For localized pustulosis in full-term neonates.
Vancomycin.	Is thought to be the best treatment for severe MRSA infections. Vancomycin inhibits the development of bacterial cell walls by targeting the sites involved in cell wall synthesis and binding permanently to the terminal d-alanyl-d-alanine of bacterial cell wall precursors.	There have been reports of VISA and VRSA infections.
Clindamycin.	Treatment of severe *S. aureus* infections. Clindamycin inhibits early chain elongation by binding to the 50S ribosomal subunit of bacteria and interfering with the transpeptidation reaction, which disrupts protein synthesis.	Use for treating SSTIs and invasive susceptible CA-MRSA infections in children, including osteomyelitis, septic arthritis, pneumonia, and lymphadenitis, despite not being specifically approved for the treatment of MRSA infections. It is not recommended for endovascular infections such as septic thrombophlebitis or infective endocarditis. Although its entry into the cerebrospinal fluid is restricted, it has exceptional tissue penetration, especially in bone and abscesses.
Linezolid.	Severe MRSA infections in newborns. Acts by preventing the 50S ribosome from initiating protein synthesis.	Approved for the treatment of nosocomial pneumonia caused by MRSA and SSTIs. Long-term use usually results in resistance through a mutation in the 23S ribosomal RNA binding site for linezolid or methylation of adenosine at position 2503 in 23SrRNA caused by the *cfr* gene.
Daptomycin.	Severe MRSA infections in newborns. Causes bactericidal action in a concentration-dependent manner by interfering with the function of cell membranes through calcium-dependent binding.	Pharmacokinetics, safety, and effectiveness in children are still being studied and have not been determined.
Rifampicin.	Exhibits bactericidal action against *S. aureus* and reaches high intracellular levels, in addition to penetrating biofilms. Rifampin inhibits DNA-dependent RNA polymerase by decreasing the RNA polymerase’s affinity for short RNA transcripts or by sterically blocking the elongating RNA’s route at its 5′ end.	It should not be used as monotherapy due to the quick development of resistance. In some situations, it may be used in conjunction with another active antibiotic.
Telavancin.	Severe MRSA infections in newborns. Prevents the formation of cell walls by attaching itself to peptidoglycan chain precursors and depolarizing cell membranes.	Should be reserved for MRSA, VISA, and VRSA.
Trimethoprim-sulfamethoxazole.	Option for the outpatient treatment of SSTIs. Inhibits dihydropteroate synthase (sulfamethoxazole) and tetrahydrofolate reductase (trimethoprim) and leads to impaired thymidine biosynthesis.	Increases the risk of kernicterus; thus, it is not advised during the first few months of life.

MRSA, methicillin-resistant *Staphylococcus aureus*; VISA, vancomycin-intermediate *Staphylococcus aureus*; VRSA, vancomycin-resistant *Staphylococcus aureus*; SSTI, skin and soft tissue infection; CA-MRSA, community-acquired MRSA.
